# Generation of Heritable Prominent Double Muscle Buttock Rabbits via Novel Site Editing of Myostatin Gene Using CRISPR/Cas9 System

**DOI:** 10.3389/fvets.2022.842074

**Published:** 2022-05-20

**Authors:** Yalin Zheng, Yu Zhang, Liyan Wu, Hasan Riaz, Zhipeng Li, Deshun Shi, Saif ur Rehman, Qingyou Liu, Kuiqing Cui

**Affiliations:** ^1^State Key Laboratory for Conservation and Utilization of Subtropical Agro-Bioresources, Guangxi University, Nanning, China; ^2^Guangdong Provincial Key Laboratory of Animal Molecular Design and Precise Breeding, School of Life Science and Engineering, Foshan University, Foshan, China; ^3^Department of Biosciences, COMSATS University Islamabad, Islamabad, Pakistan

**Keywords:** rabbits, Cas9, *MSTN* gene, knock-out, double muscle buttocks rabbits

## Abstract

Rabbits have been domesticated for meat, wool, and fur production, and have also been cherished as a companion, artistic inspiration, and an experimental model to study many human diseases. In the present study, the muscle mass negative regulator gene myostatin (*MSTN*) was knocked out in rabbits at two novel sites in exon3, and the function of these mutations was determined in subsequent generations. The prominent double muscle phenotype with hyperplasia or hypertrophy of muscle fiber was observed in the *MSTN*-KO rabbits, and a similar phenotype was confirmed in the F1 generation. Moreover, the average weight of 80-day-old *MSTN-*KO rabbits (2,452 ± 63 g) was higher than that of wild-type rabbits (2,393.2 ± 106.88 g), and also the bodyweight of *MSTN*-KO rabbits (3,708 ± 43.06g) was significantly higher (*P* < 0.001) at the age of 180 days than wild-type (WT) rabbits (3,224 ± 48.64g). In *MSTN*-KO rabbits, fourteen rabbit pups from the F1 generation and thirteen from the F2 generation stably inherited the induced *MSTN* gene mutations. Totally, 194 pups were produced in the F1 generation of which 49 were *MSTN*-KO rabbits, while 47 pups were produced in the F2 generation of which 20 were edited rabbits, and the ratio of edited to wild-type rabbits in the F2 generation was approximately 1:1. Thus, we successfully generated a heritable double muscle buttocks rabbits via myostatin mutation with CRISPR/Cas9 system, which could be valuable in rabbit's meat production and also a useful animal model to study the development of muscles among livestock species and improve their important economic traits as well as the human muscle development-related diseases.

## Introduction

Rabbits are long-eared ground-dwelling mammals belonging to the family *Leporidae* order *Lagomorpha* and domesticated about 1,400 years ago (1) due to their delicious high-quality meat. They are geographically distributed across the desert, wetland, and tropical forests (2). Rabbits are being reared at the domestic level as a livestock species for meat, wool, or fur production (3), and nowadays rabbit production has become a minor agricultural enterprise in Western European countries such as Italy, Spain, and France (1, 3, 4). The worldwide per capita rabbit meat consumption is 0.242 kg, while in some European countries like Italy the per capita consumption is about 4.39 kg (5, 6). Furthermore, a stable rate of rabbit meat consumption has also been reported from 2000 to 2013 in many European countries (5).

The body growth is primarily regulated through complex interactive pathways that cause cell proliferation and cell enlargement (7). However, several factors including hormonal, genetic, environmental, and nutritional can remarkably predict the growth patterns (8). Myostatin (*MSTN*) is a member of the transforming growth factor (TGF-β) superfamily which is considered to be a negative regulator through inhibiting muscle development and regeneration (9). *MSTN* is an extracellular hormone that occurs in the skeletal muscle in an inactive state and restores its activity by binding to the precursor peptide, follistatin 3, and TGF-β binding protein, and transmitting the signals through the receptors (9, 10). The binding affinity of *MSTN* and ActRIIB could activate a chain of signal transmission that inhibits the myocyte differentiation and proliferation (10–12).

The *MSTN* gene inactivation might have an effect on muscle development and regeneration since *MSTN* gene deletion in mice resulted in a double muscle phenomenon (hyperplasia or hypertrophy) and a significant increase in muscle mass (10–12). Natural mutations of *MSTN*, which have an obvious double muscle phenotypic effect were found in cattle (13, 14), dogs (15), sheep (16, 17), pig (18), and humans (19, 20). CRISPR/Cas9 system is an efficient genome editing tool and has widely been used for functional gene study. Knock out of *MSTN* with CRISPR/Cas9 system has been processed in sheep (21, 22), goats (23, 24), and pigs (23, 25). Qian et al. have reported the *MSTN*-KO gene in Meishan pig fetal fibroblasts by engineered zinc-finger proteins and prepared the *MSTN*-KO pig by somatic cell nuclear transfer technology. In comparison to wild-type Meishan pigs, the lean meat rate of Meishan pigs with *MSTN* knockout increased by 11.62% (26). In addition, *MSTN*-KO goats' meat production was 32% higher than the wild-type (27).

Rabbits offer quick breeding sources and meat with excellent nutritive and dietetic properties which contain high-quality protein and low fat and cholesterol contents, that is fine-grained white meat which can substitute chicken, and is also ideal for obese and cardiovascular patients (28). In the present study, we have designed a highly efficient novel regulatory site and additionally verified that the exon 3 region can regulate the *MSTN* gene. Furthermore, in our study, the rabbits obtained after the *MSTN* gene editing are able to pass the gene-edited traits to the next-generation normally, and homozygous gene-edited rabbits obtained in the F2 generation have normal production performance and stable double muscle buttock characteristics.

## Materials and Methods

### Animals

In the present study, rabbits were used to perform all the experiments according to the Principle Guideline for the Use and Care of Laboratory Animals, Guangxi University. Rabbits were kept under controlled conditions at the Animal Center of Guangxi University. A total of 33 rabbits used for ovulation were approximately 6–8 months old and weighed between 3.5 and 4.5 kg.

### SgRNA Design and Plasmid Construction

**Figure 1:**
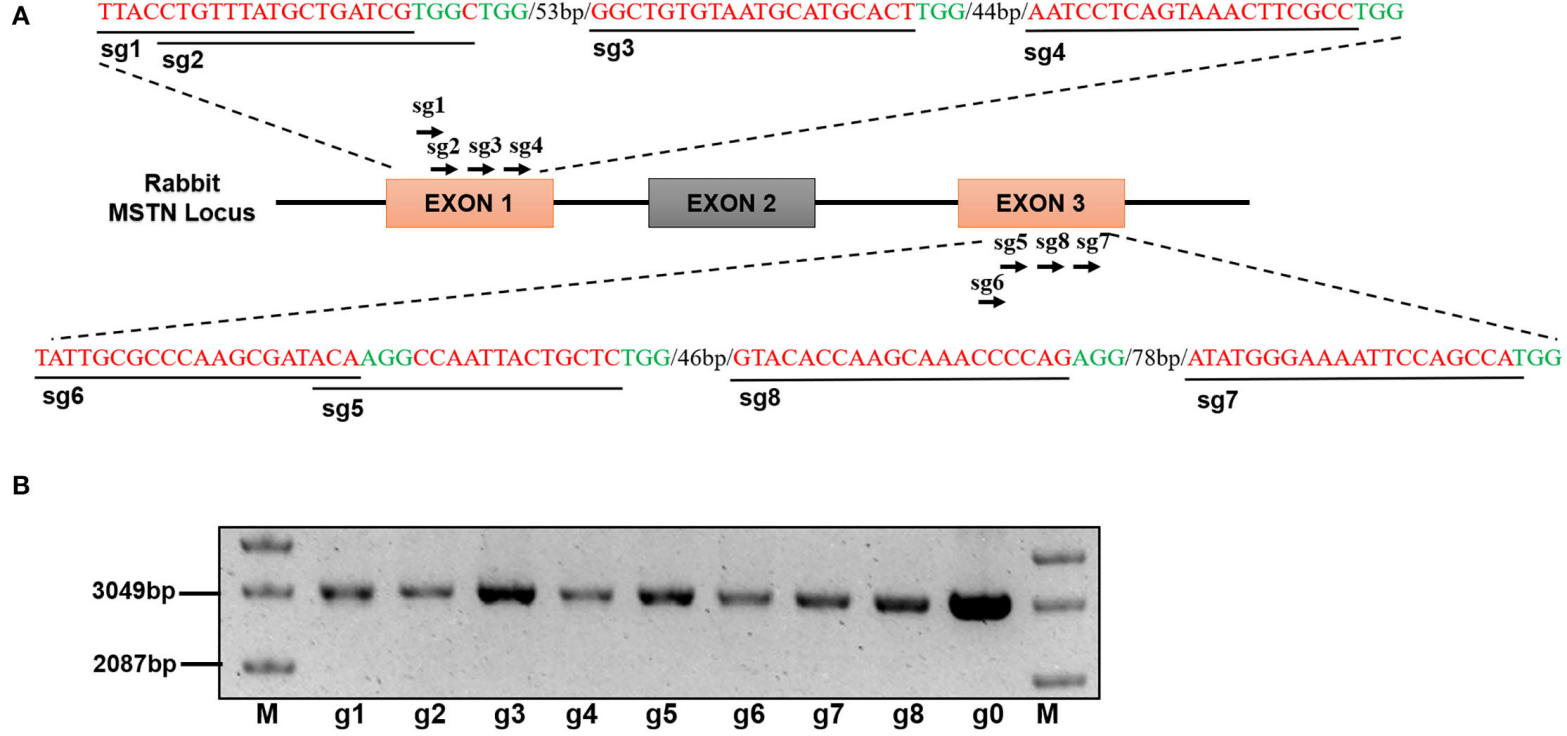
**(A)** Design and construction of sgRNA Schematic diagram of sgRNA targeting site in the rabbit *MSTN* gene locus. **(B)** Electropherogram of eight sgRNA vectors, M: Supercoiled DNA Ladder Marker, g1-g8 refer to phU6-gRNA-Sg1to phU6-gRNA-Sg8 (The picture has been cropped).

A total of 8 sgRNAs *MSTN* sites were designed using the website (http://www.genome-engineering.org/) and named as g1, g2, g3, g4, g5, g6, g7, and g8 (Figure 1A). The complementary DNA strand was annealed to develop into a double strand and cloned into the pMD18-hU6-gRNA vector. Primers T76 and T78 were used as marked gene fragments and amplified by targeting two sgRNA vectors. Linearized DNA plasmids and PCR products were extracted and purified using the MEGA shortscrip TM T7 kit (Ambion, USA). The sgRNAs were generated according to the manufacturer's recommendations.

### Fibroblasts Culturing and Electroporation

Primary rabbit fibroblasts were cultured in 10% (v/v) FBS, 35 mm glutamine, and 1 × DMEM (Gibco). The plasmid was transfected into fibroblasts through electroporation (Gene Pulser Xcell & BIO-RAD) at a pulse of 225 volts for 10 ms. Briefly, 4 μg of pCMV-T7-NLS-hSpCas9-NLS (hSpCas9) DNA plasmid and 2 μg of gRNA plasmid were used to transfect 5 × 10^5^-10 × 10^5^ fibroblasts. After 24 h the culturing media was changed to DMEM containing 10% FBS. Subsequently cultured for 4 days, the DNA was extracted and the *MSTN* was amplified by PCR and sent to the Beijing Genomics Institute (BGI) for sequencing to analyze the editing effect of the gene fragment. Optimal sgRNA was selected for the following experiment.

### Zygote Collection and Blastocyst Genotype Testing

Rabbits (*n* = 3) in estrus were mated, the female rabbits were anesthetized 16–20 h after mating, and the fertilized eggs were collected from the oviduct with a cell culture medium consisting of M199 (Gibco, USA), 3% bovine serum (Gibco, USA), 5 mM HEPES (Sigma, USA), and 5 mM NaHCO3 (Sigma, USA). Then, the zygotes were microinjected with Cas9 protein and sgRNA and the blastocysts were cultured *in vitro*. The mutation of the target region was analyzed by PCR and T7EI digestion assay.

### Generation of Transgenic Rabbit

The zygote cytoplasm was injected with a 10-pl CRISPR/Cas9 mixture containing 200 ng/μl Cas9 protein (A36497, Hot Fisher, USA), 20 ng/μl sgRNA6, and 20 ng/μl sgRNA8, and the zygotes were transferred back to the oviduct of the recipient rabbit (*n* = 30). The pregnancy phenomenon was checked 10 days after the transplantation and the pups were born after 30 days.

### T7 Endonuclease I (T7EI) Assay

Rabbit ear tissue genomic DNA was extracted using an extraction kit (Quick-DNA Plus, ZYMO RESEARCH) and CRISPR/Cas9-induced mutations were studied by PCR and T7E1 assay. PCR was performed using primers flanking the target site and the product was sequenced (Shanghai Sangon Company, Shanghai, China) and digested with T7EI (NEB, USA) to characterize the mutation.

### Off-Target Analysis

Potential off-target sites of the sgRNAs were assessed using the CRISPR design tool (http://crispor.tefor.net/) and the top 4 potential off-target sites of each sgRNA were selected and amplified by PCR with specific primers (Table 1). PCR products were evaluated by sequencing and T7E1 digestion assay.

**Table 1:** Primers of off-target detection.

**Primer**	**Sequence (5^′^→3^′^)**	**Length (bp)**
POTs6-1F	CAATGGTGTGAGCCTCAAAG	382
POTs6-1R	AGTGGTCGTCTTCTTCATCC	
POTs6-2F	ATGCTCCTGTGTAGTCACTG	373
POTs6-2R	GTTTTCCATGTCCAGCTCAC	
POTs6-3F	ATCCAGGTATTAGCAACCGT	292
POTs6-3R	TCTGTGAATGTGCATACATACA	
POTs6-4F	TGACTACTGGCCCAAAATGT	450
POTs6-4R	TTCAGTCACAGAGTCGGTTT	
POTs6-5F	GAAGGGCCACAAAGAGAAAG	238
POTs6-5R	AAGGCCTCTTCCTCCCAG	
POTs8-1F	GGCGCTCATGATCTCTTGCT	291
POTs8-1R	CCTCACCAATGTCGATGCCT	
POTs8-2F	AGCAGACATTCTGGCGGAAA	348
POTs8-2R	GCCAAATGCAGCCTCAGAAT	
POTs8-3F	TCCAGAGTGCCTGCAGGTA	351
POTs8-3R	TCCAGAAGCTCAAATCTCTTGCT	
POTs8-4F	GTTTGGACACTGCTTGCTGG	291
POTs8-4R	TCATGGTGGATGCCCTCTTG	
POTs8-5F	CACACACATCCTCGGCTCAT	352
POTs8-5R	CATTACCTTCCTCCCACCCC	

### Western Blot Analysis

*Gluteus maximus* tissue samples were collected from 5 *MSTN*-KO and 5 WT rabbits (anesthesia sampling at 6 months of age), the tissues were ground in liquid nitrogen and 2.5 μl/ml protease inhibitor was added and kept in ice for 30 min. Protein concentration was determined using the Bradford method (Bio-Rad), and 35 μg of protein sample was subjected to a 5–12% separation by SDS-polyacrylamide gel. Anti-MSTN polyclonal antibody (Abcam) and goat anti-rabbit IgG were coupled to horseradish peroxidase (HRP; Santa Cruz, USA) antibodies for protein detection, while β-actin antibody (Santa Cruz) was used as an internal control.

### Sample Collection, Physical and Histological Analysis

A total of 10 rabbits, including 5 F0 *MSTN*-KO and 5 WT, were fed at the same conditions and weaned on the 30th day. Rabbits were kept in separate cages and their body weight was recorded after every 10 days for 200 days. At the age of 6 months, the rabbits were anesthetized with pentobarbital sodium (1 mL/kg) to take gluteal muscle mass tissue. The gluteus maximus tissues of the F0 generation *MSTN*-KO and WT rabbits were fixed with 4% paraformaldehyde at 4°C. Different concentrations of dehydrated sucrose (30% for 7 h, 40% for 7 h, 45% for 5 h) were also used and frozen at −25°C for histological examination. The tissue slices of 5 μm thickness were first stained with hematoxylin and eosin (H&E) and then examined by a fluorescence inverted microscope (Nikon, Japan). The integral optical density (IOD) analysis of histological sections was performed by using Image-Pro Plus 6.0 software.

### Growth Data Recording of Edited Rabbit F1 and F2 Generations

The F0 generation edited rabbits were bred with wild type to obtain the F1 generation rabbits with the edited genotype. There were 3 male parents of edited rabbits and 22 wild-type female rabbits. All the F1 generation-produced edited rabbits were well developed and had significant differences in body weight from the wild type and were reserved for seed use. After sexual maturity, two breeding methods were adopted for breeding with the same genotype, and the edited male rabbits were bred with wild-type female rabbits to produce the F2 generation. Among them, there were 4 male-edited rabbits, 5 female-edited rabbits, and 9 wild-type female rabbits of the F1 generation. Genotype identification was carried out and two genotypes insertion type and deletion type were selected. The F2 generation edited rabbits were crossbred with the same genotype and also with the wild type. The weight recording method is consistent with the F0 generation edited rabbit recording method. Among them, the breeding and farrowing information of the F1 generation male rabbits were recorded.

### Statistical Analysis

All the data obtained in this study were analyzed using Graph pad prism software (*T*-test) and the *p* < 0.05 was considered statistically significant (29).

## Results

### The SgRNA Designing and Construction

The exon 1 and 3 of the *MSTN* gene were targeted to design eight sgRNAs (g1, g2, g3, g4, g5, g6, g7, and g8) which were cloned into the pMD18T vector as shown in Figure 1A. The recombinant vector was named pMD18-hU6-gRNA and the sequence analysis results, as presented in Figure 1B, confirmed that the sgRNA has been successfully inserted into the vector.

### Mutational Effect of the SgRNAs in Rabbit Fibroblasts

**Figure 2:**
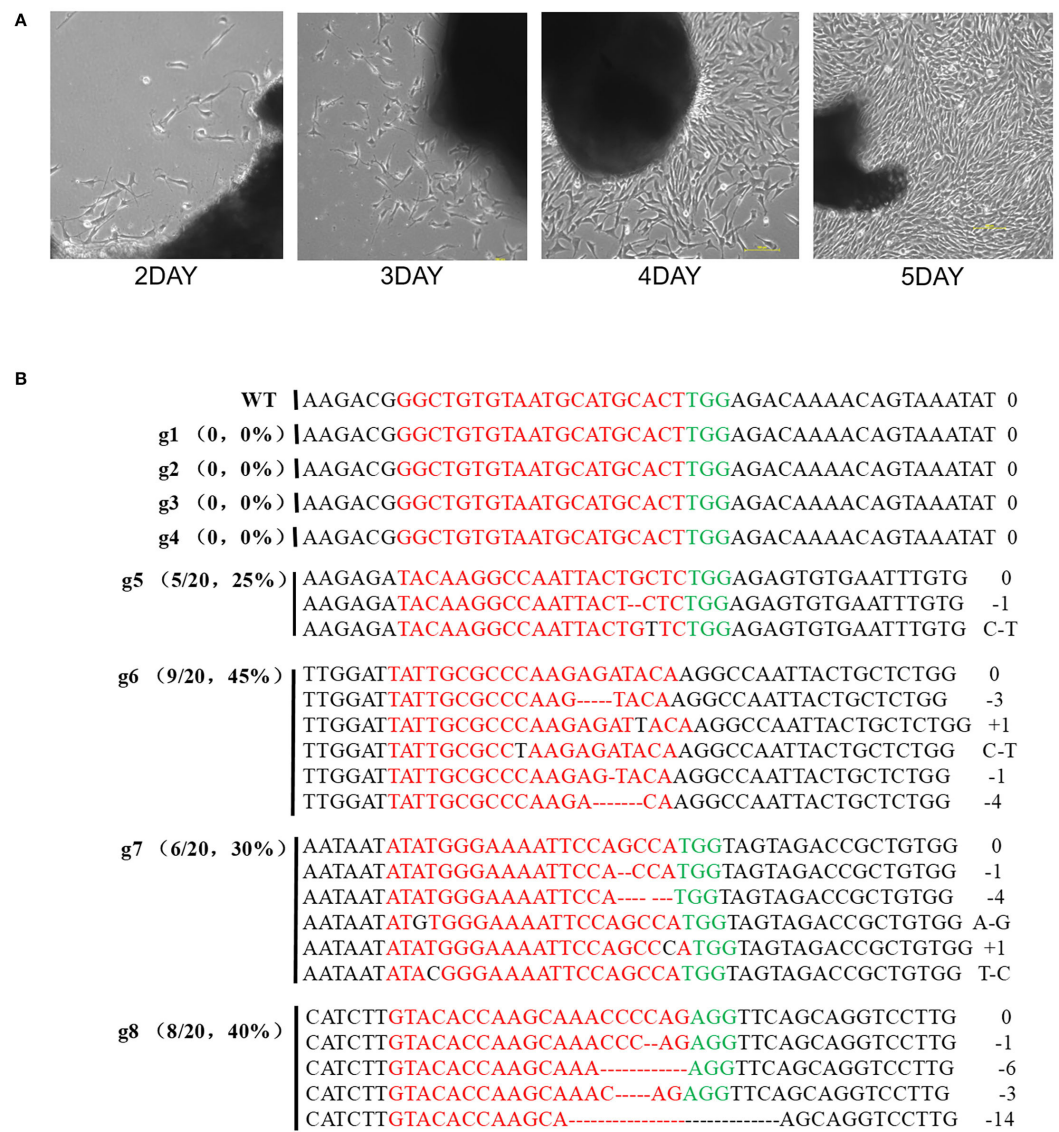
The results of sequencing for mutations at the specific sites in rabbit REFs. **(A)** Rabbit Primary fibroblast cell in culture from 2days to 5days. **(B)** Sanger sequence analysis of REFs. g1-g4 target to exon1, g5-g8 target to exon 3. gRNA edited were named on the left. g1-g8 represent different gRNA, PAM sequence is marked in green. The sgRNAs sequence is marked in red. The numbers on the right indicated the type of mutation, with “-” represents deletion of the given number of nucleotides, “+” represents insertion of the given number of nucleotides.

The rabbit primary fibroblasts were obtained from newborns (Figure 2A) and the mutation efficiency of the designed sgRNAs was confirmed. In g1, g2, g3, and g4 no mutations were generated while the rate of mutation in g5, g6, g7, and g8 was observed to be about 20–45% (Figure 2B). Further, the sgRNAs targeted to exon 3 were significantly better than that of exon 1. The gene alteration proportion of sgRNA6 (45%, 9/20) and sgRNA8 (40%, 8/20) were higher than that of the other gRNAs. Thus, sgRNA6 and sgRNA8 were selected for further experiments.

### CRISPR/Cas9-Mediated MSTN Site-Specific KNOCK-OUT in Rabbit Zygotes

**Figure 3:**
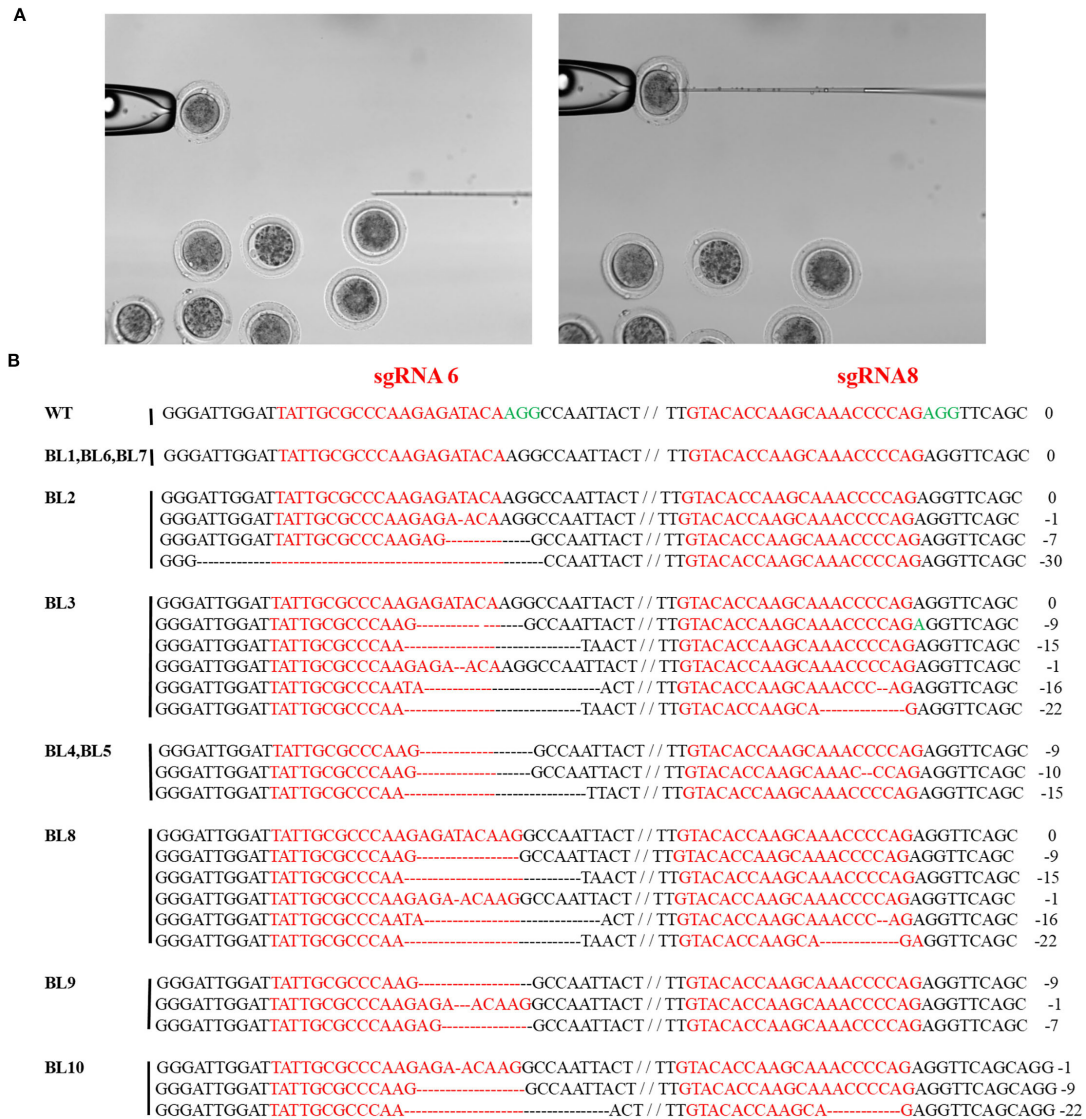
Targeted mutagenesis efficiency of the CRISPR/Cas9 system in rabbit zygotes. **(A)** Schematic diagram of the microinjection of Cas9 mRNA and sgRNAs into rabbit zygotes. **(B)** BL1-BL7 represent different blastocysts used in this study. Sanger sequence analysis of zygotes. zygotes edited were named on the left. PAM sequence is marked in green. The sgRNAs sequence is marked in red. The numbers on the right indicated the type of mutation, with “-” represents deletion of the given number of nucleotides.

The CRISPR/Cas9 system containing sgRNA6 and sgRNA8 was injected into the zygotes to verify the site-specific deletion of the *MSTN* gene. A total of 16 zygotes were used for injection purposes, of which 10 developed to the blastocyst stage through *in vitro* maturation (Figure 3A). The genome-editing efficiency in the blastocysts was examined by PCR and Sanger sequencing, and the results revealed that 70% (7/10) of the blastocysts had genetic mutations (Figure 3B).

### Generation of MSTN Knock-Out Rabbits

**Figure 4:**
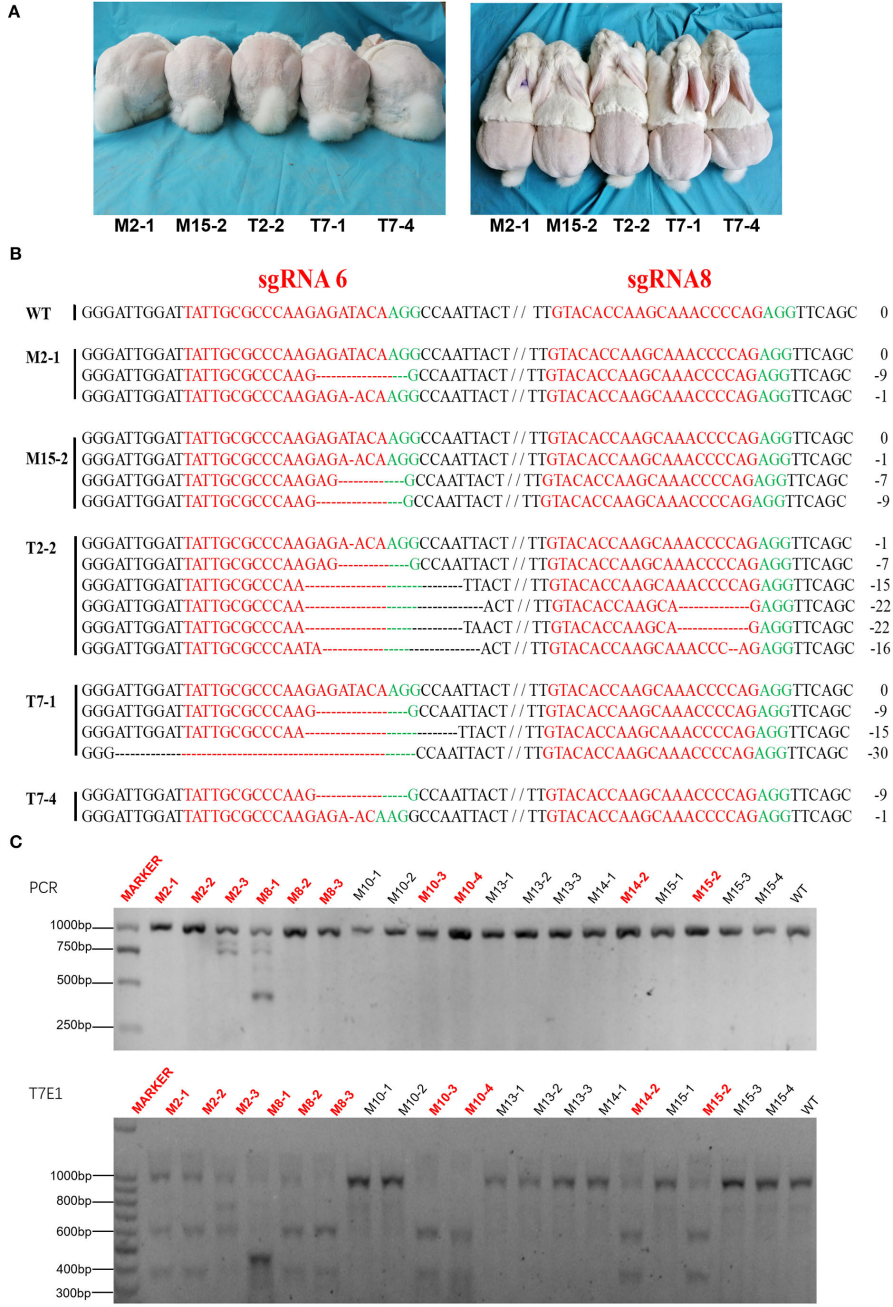
Generation of F0 *MSTN* KO rabbits via CRISPR/Cas9. **(A)** Five mutated rabbits of F0. **(B)** Sanger sequence analysis of mutated rabbits. Five rabbits edited were named on the left. WT stands for wild type control; PAM sequence is marked in green. The sgRNAs sequence is marked in red. The numbers on the right indicated the type of mutation, with “-” represents deletion of the given number of nucleotides. **(C)** Upper: The results of sequencing for mutations at the specific sites in Agarose gel electrophoresis of PCR products about a part of pups of F0. Lower: T7E1 cleavage assay for the rabbits (The picture has been cropped).

A total of 99 cytoplasmic injected zygotes were transplanted to the oviduct of 10 female receptor rabbits, and 23 rabbit pups were born after full-term gestation (Table 2). Gene-editing was evaluated by T7E1 digestion and Sanger sequencing analysis which presented that 5 of them were *MSTN* knock-out rabbits (Figure 4) and interestingly, three *MSTN*^+/−^ rabbits were all males, while two *MSTN*^−/−^ rabbits were all females. The further T7E1 and sequencing analysis showed that 5 *MSTN* knock-out rabbits were chimeras.

**Table 2:** Generation of the *MSTN*-KO rabbits via CRISPR/Cas9.

**No**.	**SgRNA**	**gRNA/Cas9 protein (ng/uL)**	**Embryos injected**	**Embryos transferred**	**Pregnancy**	**Pups obtained (%transferred)**	***MSTN* KO pups (%pups)**
M02	Sg6+Sg8	40/200	14	14	Yes	3 (21.40%)	1 (33.33%)
M08	Sg6+Sg8	40/200	12	12	Yes	3 (25.00%)	1 (33.33%)
M09	Sg6+Sg8	40/200	7	7	Yes	4 (57.14%)	0 (0)
M10	Sg6+Sg8	40/200	10	10	Yes	4 (40.00%)	2 (50.00%)
M12	Sg6+Sg8	40/200	9	8	Yes	4 (50.00%)	1 (25.00%)
M13	Sg6+Sg8	40/200	8	8	Yes	3 (37.50%)	0 (0)
M14	Sg6+Sg8	40/200	7	14	Yes	2 (14.29%)	1 (50.00%)
M15	Sg6+Sg8	40/200	9	7	Yes	4 (57.14%)	1 (25.00%)
M17	Sg6+Sg8	40/200	11	11	Yes	4 (36.36%)	2 (50.00%)

### Off-Target Mutation Analysis of *MSTN* Knock-Out Rabbits

**Figure 5:**
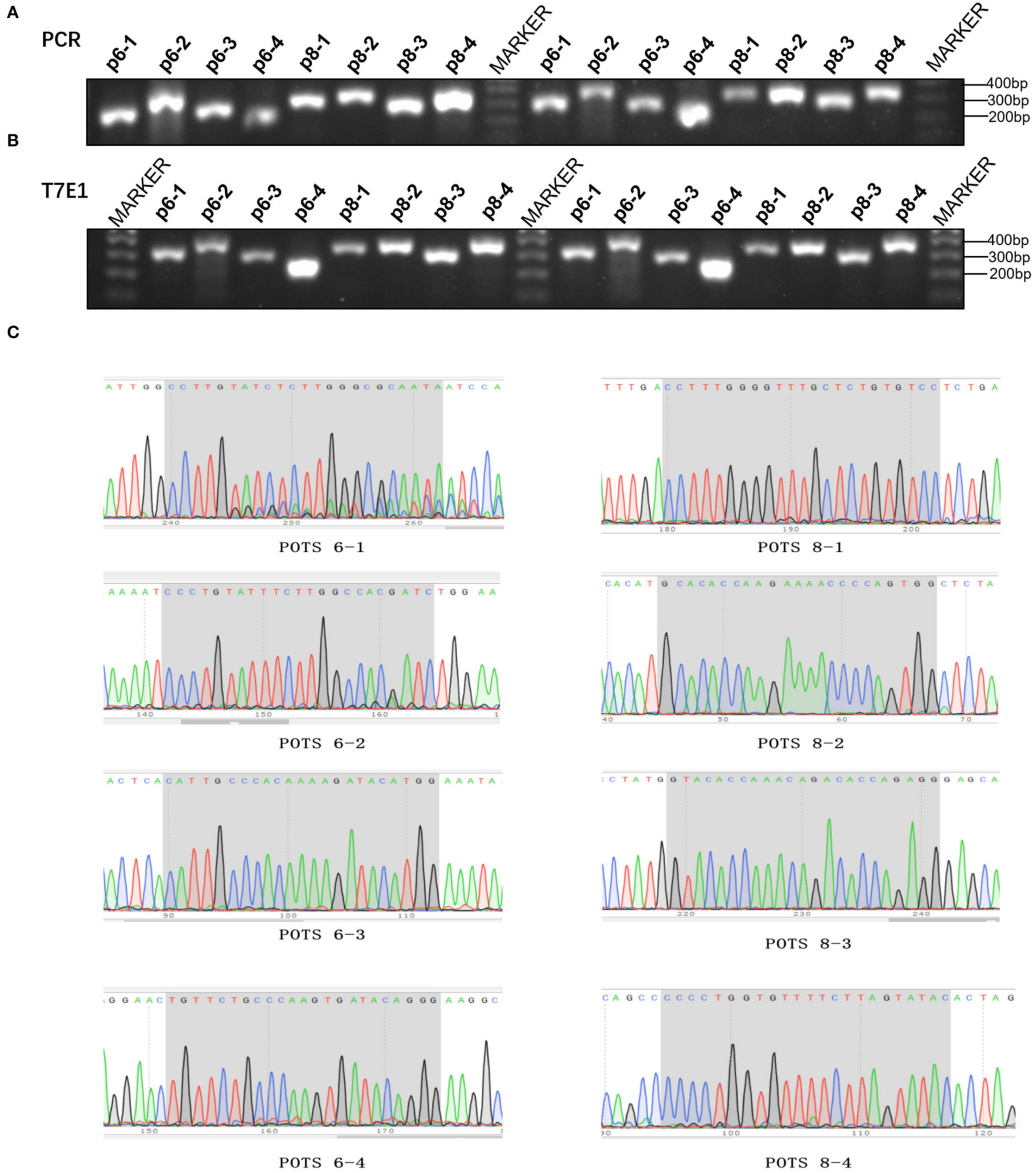
Detection of *MSTN* modified rabbits. **(A)** The results of suspicious POTs in Agarose gel electrophoresis of PCR products. M: DL100bp; p6-1–p6-4 represent g6 four suspicious POTs, p8-1–p8-4 represent g8 four suspicious POTs (The picture has been cropped). **(B)** The results of suspicious POTs in Agarose gel electrophoresis of T7E1. **(C)** Sanger sequencing for suspicious POTs sites.

To detect the presence of off-target mutations in *MSTN* knockout rabbits, four potential off-target sites were designed. The T7EI digestion and Sanger sequencing results exhibited that no off-target mutation was detected in the *MSTN*-KO rabbits (Figure 5).

### Western Blot Analysis of *MSTN*-KO Rabbits

**Figure 6:**
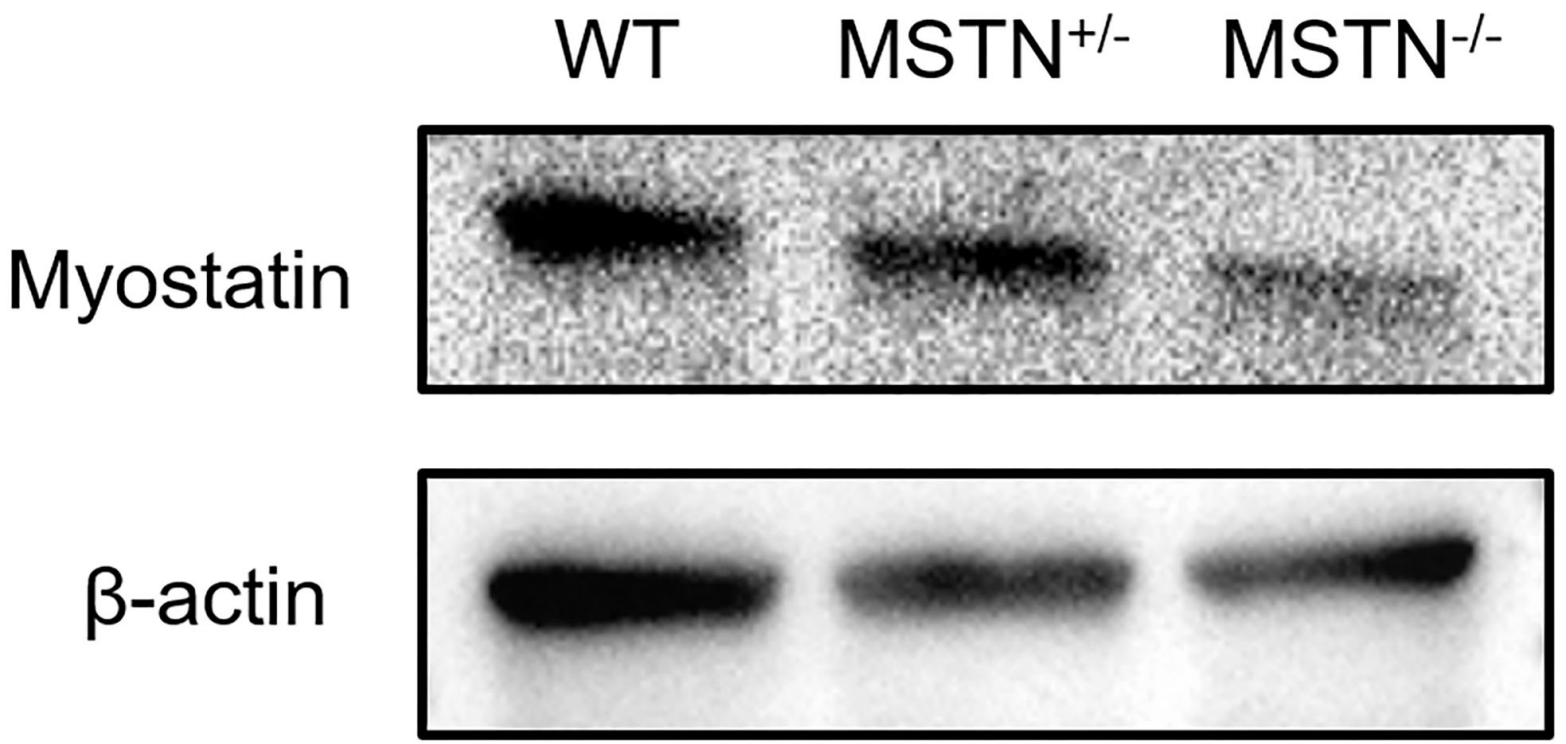
Result of *MSTN*+*/–, MSTN–/–* and WT rabbits in Western Blot. Beta-actin served as a loading control (The picture has been cropped).

According to the results of western blot analysis, it was revealed that the expression level of *MSTN* protein in the muscle of mutant rabbits was lower than that of WT rabbits (Figure 6).

### Morphological Analysis of *MSTN*-KO Rabbits

**Figure 7:**
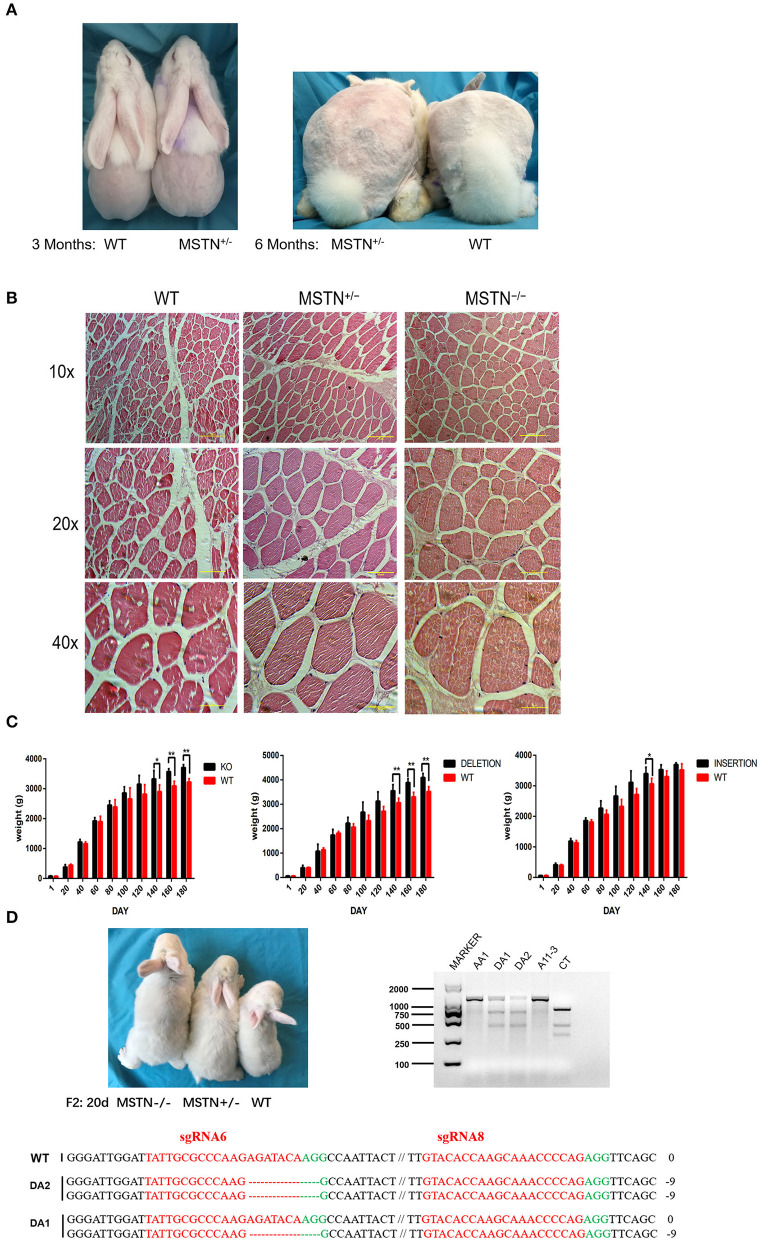
Detection and observation of *MSTN*-KO rabbits. **(A)** Comparison of mediated and wild-type rabbits at 3 and 6 months of age. **(B)** The average body weight of *MSTN*^+/−^ and WT rabbits from F0 and F1 (*n* = 5), and the left side shows the weight gain of F0 knockout rabbits and wild rabbits, the middle shows the weight gain of F1 deletion editing rabbits and wild rabbits, and the right side shows the weight gain of F1 insertion editing rabbits and wild rabbits. **(C)** HandE staining of the muscle fibers from *gluteus maximus*. **(D)** Shape difference map and identification results of F2 generation rabbits at the age of 20 days (The picture has been cropped).

The appearance of WT and F0 generation edited rabbits at the age of 3rd and 6th months were observed (Figure 7A). Under the same feeding conditions, the *biceps femoris* was more developed in the F0-generation edited rabbits than the WT. Moreover, the difference in muscle development of the *MSTN-*KO rabbit was evaluated using body weight, and data related to weight gain is shown in Supplementary Table S1. No significant difference was observed related to the body weight in the five *MSTN-*KO rabbits and the control group within the early 90 days (*p* > 0.05). But after growing to the age of 3rd month, the five *MSTN*-KO rabbits' weight began to appear slightly higher than that of the control group (Figure 7B) and the “double muscle” phenomenon appeared more obvious in *MSTN*-KO rabbits. At the age of 140 days, the five *MSTN*-KO rabbits exhibited a significantly higher body weight than the WT control group rabbits (*P* < 0.05, *n* = 5). Furthermore, the bodyweight of *MSTN*-KO rabbits (3,572 ± 41.40 g) was significantly (*P* < 0.01) more at 160 days as compared to the control WT rabbit (3,094 ± 69.54 g). Additionally, the weight gain data from two selected genotypes (insertion and deletion type) of the F1 generation rabbit are shown in Supplementary Table S2. A significant weight gain has been observed for both the insertion type and the deletion type in comparison to WT at 140 days, where the bodyweight of insertion type rabbits was 3,398 ± 92.97 g, deletion type rabbits 3,554 ± 110.45 g, and WT rabbits 3,064 ± 80.35 g (*P* < 0.05, *n* = 5), respectively. The *gluteus* of the *MSTN*-KO rabbits was also bigger than the WT rabbits, and histological analysis showed that in *MSTN*-KO rabbits the density and diameter of the myofiber were significantly large (Figure 7C). Also, it was found that the phenotypic differences of the edited rabbits were stably inherited in the F2 generation, and Figure 7D shows three young rabbits in the same litter. Similarly, T7E1 enzyme digestion and sequencing identification was used for young rabbit genotyping, and it has been observed that the body size of the edited rabbit was significantly different from that of the WT.

### Heritability of the *MSTN*-KO Rabbits

**Figure 8:**
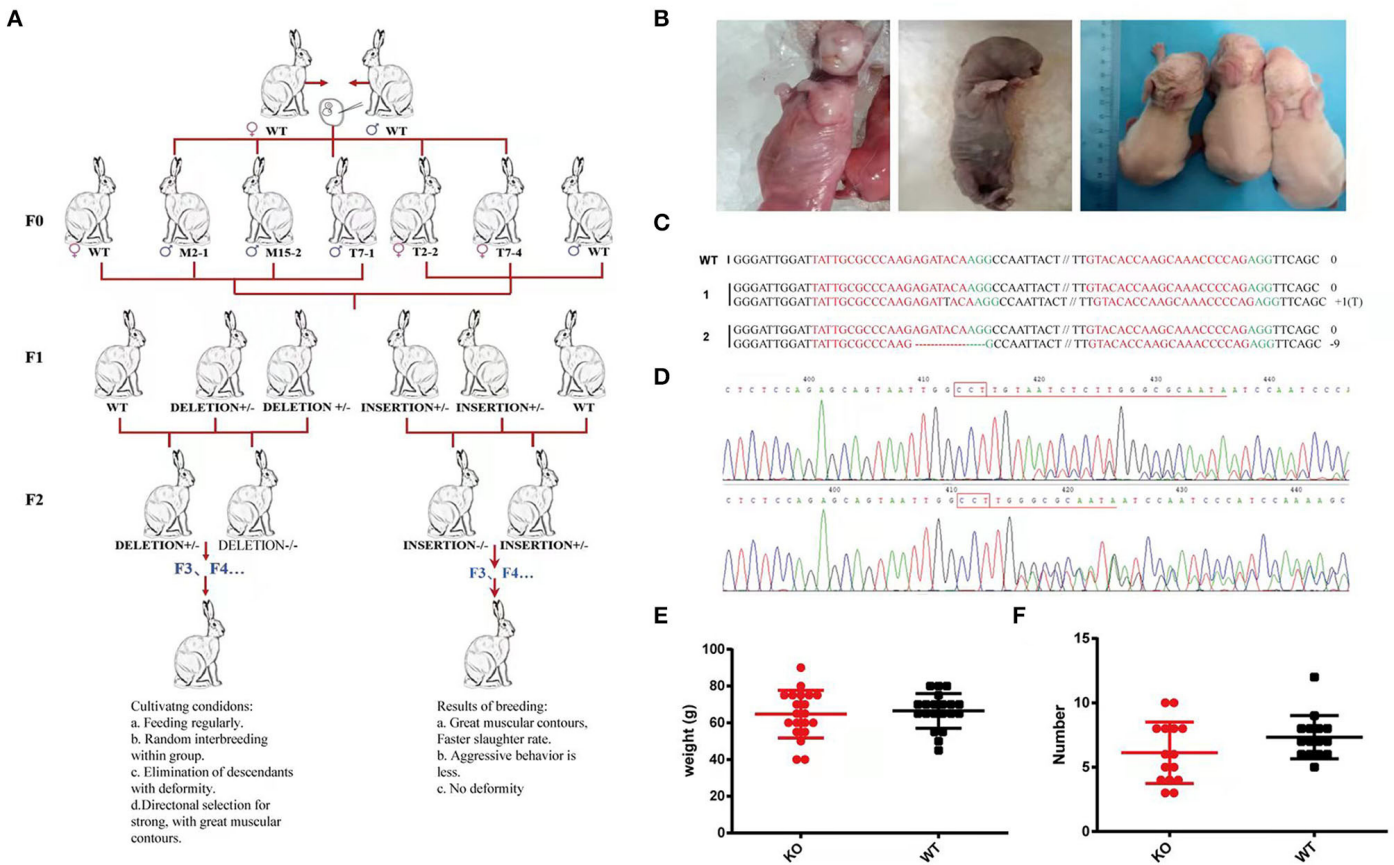
**(A)** Schematic diagram of *MSTN*-KO rabbits breeding family. **(B)** Left: A cub with a large tongue. Right: Difficult to give birth due to the large size of the cubs. **(C)** Rabbits of F1 were named on the left. 1-2 represent different rabbits, PAM sequence is marked in green. The sgRNAs sequence is marked in red. The numbers on the right indicated the type of mutation, with “-” represents deletion of the given number of nucleotides, “+” represents insertion of the given number of nucleotides. **(D)** sequence identification. **(E)** Birth weight of rabbits. **(F)** Litter size.

To determine whether the offspring could stably inherit the successfully edited gene fragments from the *MSTN*-KO, all the F0 generation edited rabbits were crossed with wild-type rabbits. The three *MSTN*-KO F0 male rabbits were mated with wild rabbits and live F1 rabbit pups were produced. The T-cloning sequencing and T7E1 cleavage assay demonstrated that 28 out of the 53 newborn F1 rabbits carried *MSTN* mutations, of which 23 were monoallelic, and 5 were biallelic *MSTN*-KO rabbits (Figure 8). Two genotypes, deletion type and insertion type, were selected from the F1 generation to continue breeding, and the edited rabbits of the F2 generation were obtained. There was no significant difference in the litter size of female rabbits after breeding between F1 generation male rabbits and wild-type female rabbits, and there was no significant difference in the birth weight of offspring (Figure 8). So, there were three homozygous edited rabbits at the age of 1 month, with obvious differences in appearance and body shape. As shown in Figure 8, it is expected that a small number of homozygous edited rabbits obtained from the F2 generation, and in the F3 generation these edited homozygous rabbits continue to produce until a stable expansion thereof be achieved.

## Discussion

Humans are entirely reliant on livestock for their daily food supply, which comes in the form of eggs, meat, and milk (30–33). Thus, genetic alteration offers an opportunity for significant production and gains in a short time (34, 35). Since the last decade, many gene-edited organisms have been approved and are being used by the public, such as the gene-edited goats' milk and chicken eggs are being used for drug extraction. Further, the salmon was the first gene-edited species to be approved to be consumed as food, and recently the GalSafe pigs have also been certified to be used for food and medical purpose (36). Rabbit farming is now very well developed all around the world and systematically reared on a large scale, and the global rabbit meat production is reaching 1.8 million metric tons per year (37). As high-quality meat, the demand for rabbit meat is worth looking forward to in the future, but rabbits' muscular development is still a challenge. Therefore, *MSTN* gene-edited rabbits have the possibility of being approved and used by the public under the advancement of our multi-generation continuous observational research. In the present study, we used CRISPR-Cas9 to delete a long gene fragment of the *MSTN* gene, which ensured the inactivation of target gene function, and the results of our study presented a successful production of *MSTN-*KO rabbit with heritable ability, and the loss of the *MSTN* fragment could lead to muscle growth. In both the F0 and F1 generations, there were significant differences in body weight before it reached the plateau, and the average body weight of the edited rabbits before the slaughter age was also higher than that of the WT rabbits.

Although the increase in muscle mass makes *MSTN*-KO livestock production more attractive, in these *MSTN* mutant animals calving difficulties are often caused by various reasons, such as large offspring syndrome (LOS) has been reported in *MSTN*-deficient animals (15, 38). Compared with other livestock, *MSTN* gene-edited rabbits rarely report dystocia due to large fetuses, even though in our study the *MSTN*-KO rabbits exhibited a typical double-muscle phenotype with increased body weight but, at birth, no significant differences have been observed in body weight and size as compared with the control WT. Further, the *MSTN*-KO rabbits appeared healthy and normal without reproduction difficulties, demonstrating that CRISPR/Cas9 system-generated *MSTN*-KO rabbits are best suited for studying muscle development and associated diseases, and the results of our study are in line with those of Lv et al. (39).

*MSTN* gene can code for TGF-beta (transforming growth factor-beta) superfamily ligand that can bind with different TGF-beta receptors and ultimately recruit or activate the SMAD family transcription factors, which regulate the gene expression (40). *MSTN* is a key regulator of muscle cell proliferation and differentiation throughout muscle development, and *MSTN* expression signals were detected from the early myogenic stage of embryonic sarcomere formation to the adult skeletal muscle development stage (14, 41, 42). Earlier, the birth weight of *MSTN* mutant animals has also been reported in sheep, cattle, and goats (43, 44). Wang et al. have also described the *MSTN* gene-modification and their effects in goats with larger muscle fiber size resulting in enhanced bodyweight (43). Therefore, the *MSTN* gene knockout might affect muscular development at the embryonic stage (43, 44). Furthermore, it has been stated that *MSTN* can only express in skeletal muscle during fetal development, thereby controlling the differentiation and proliferation of myoblasts (43, 44). In our study, *MSTN*-KO rabbits exhibited a typical double-muscle phenotypic trait, but there was no significant difference in body size at birth (*P* > 0.05) and body weight in the first 60 days compared with the WT group. This might be because of the IGF-1 which is an important positive regulator of muscle cell proliferation and differentiation in the skeletal muscle (45). The IGF1 signaling pathway is critically involved in long-term health regulation, which ultimately plays an essential role to control various homeostatic mechanisms related to growth or development (46). Despite the inhibition of *MSTN* signal expression, IGF signaling still might upregulate the expression of myostatin in skeletal muscle tissue models, which indicated the presence of an autoregulatory inhibitory loop in a muscular system (47, 48).

Moreover, the muscle fiber hypertrophy, hyperplasia, or a combination thereof triggered by the lack of the *MSTN* gene subsequently results in increased mammalian muscle production (49–52). The quantity of muscle fibers is mainly determined before birth because the diameter of muscle fibers might be increased after birth (53, 54). The mechanism of muscle hypertrophy and hyperplasia may not occur simultaneously, and it appears that the mechanism by which *MSTN* mutations enhance the muscle mass varies among species (55, 56). In *MSTN* mutant mice and goats, the muscle mass gain was caused by muscle fiber hyperplasia and diameter hypertrophy (39, 52, 57), while only muscle fiber hyperplasia was observed in *MSTN* mutant cattle and pigs (13, 50). The results of the current study presented that the *gluteus maximus* of the *MSTN*-KO rabbits was also bigger than the WT rabbits, and histological analysis showed that in *MSTN*-KO rabbits the density and diameter of the myofiber were significantly large. Therefore, we hypothesized that the enhanced muscular phenotype in the *MSTN*-KO rabbits is attributed to muscles fiber hyperplasia and/or hypertrophy. Furthermore, previously it has been reported that the muscle mass of *MSTN*-KO mice was 2–3 times more than that of the WT mice, which was the result of the combined action of muscle fiber hyperplasia and diameter hypertrophy (13, 52). On the other hand, the cattle with double-muscle trait showed that muscle mass increased by 20–25%, and the increased muscle weight appears to be the result of muscle fiber diameter proliferation, rather than an increase in muscle number (13, 50). In this study, compared with WT rabbits, the average body weight of F0 and F1 generation edited rabbits increased by 15 and 15.99% at the age of 180 and 140 days, respectively. The fragment deletion targeting a specific site in *MSTN*-KO rabbits could suggest that the sgRNA-based CRISPR/Cas9 system might be a useful tool for gene knockout in the mammalian genome (58). Earlier in rabbits, Lv et al. (39) designed two knockout sites at the first exon to edit the *MSTN* gene (39), while we designed a total of 8 sites in two exons (exon 1 and exon 3) each with four sites. After selection, we determined that the knockout efficiency of the third exon is better than the first exon, thus, we obtained edited rabbits with different knockout sites from already reported *MSTN* knockout studies. Moreover, at present, most of the experimental research regarding animals' gene-editing is still in the F0 generation (24, 59, 60), including the reported gene editing in rabbits (39). While, no study has yet been performed on the F1 or their subsequent generations, we not only developed *MSTN* gene-edited rabbits in the F0 generation but also continued the follow-up research in successive F1 and F2 generations with stable heritable mutant populations. Also, the difference between the body type of mutant and the wild type can be observed within 1 month after birth. Gene-editing technology can accelerate the breeding process and improve the meat production of livestock, but the congenital defects of gene edited animals and the uncertainty of trait stability have a great impact on the commercialization of edited animals (51, 61). In our study medium- and long-term breeding can greatly reduce concerns about the phenotypic and genetic stability of *MSTN* gene-edited animals.

Moreover, various studies reported different types of skeletal-related problems in gene-edited animals such as *MSTN* gene-edited rabbits had bone deformities, pelvic tilt, and tooth dislocation, while *MSTN* gene knock-out Meishan pigs had an extrathoracic vertebra (26, 37). Abnormal pelvic structure may cause difficulties in calving in female animals. But, in our study, the *MSTN* gene-edited rabbits obtained by knocking out the third exon did not show these symptoms in appearance, which are more suitable for the breeding of strains. Additionally, in the previous study, the *MSTN*-KO animals exhibited the muscular feature, but the *MSTN*-KO caused severe health issues, such as the fetus having “large tongue syndrome,” which results in milk suckling problems and death (52). In our study, the *MSTN*-KO rabbits not only exhibited double mushy buttocks but were also able to inherit this trait to subsequent generations without any problem. The majority of *MSTN*-KO rabbits showed a rapid increase in muscle mass after the age of 60 days, and the *MSTN*-KO rabbits were healthy and could inherit the knocked-out gene fragments, demonstrating that the *MSTN*-KO rabbits produced by the CRISPR/Cas9 system are suitable for studying muscle development and related diseases.

## Conclusion

The rabbit meat is an enriched source of minerals with low fat and calories and is easy to digest, making it valuable for the consumers, especially for persons suffering from cardiovascular diseases or obesity. Taken together, we developed *MSTN*-KO rabbits with a typical phenotypic trait of double muscle buttocks that are likely to obtain edited rabbits lines which would help to improve the rabbit's meat production efficiency and promote the development of the rabbit industry. Furthermore, these *MSTN*-KO rabbits could be a promising tool for studying the development of muscles in other livestock species and improving their important economic trait.

## Data Availability Statement

The datasets presented in this study can be found in Genbank repository accession number MZ427879-MZ427887 at: https://www.ncbi.nlm.nih.gov/nuccore/.

## Ethics Statement

The animal study was reviewed and approved by Experimental Animal Care and Use Committee of Guangxi University (Permit Code: 2022-GXU-006). Written informed consent was obtained from the owners for the participation of their animals in this study.

## Author Contributions

SR, KC, and QL: conceptualization and resources. YZ, YLZ, and LW: data curation. YLZ, YZ, and ZL: methodology. HR, ZL, and DS: software. QL and KC: supervision. YZ and YLZ: writing—original draft preparation. YLZ, YZ, HR, ZL, DS, SR, QL, and KC: writing—review and editing. All authors have read and agreed to the published version of the manuscript.

## Funding

The present study was granted and supported by the National Natural Science Fund (Grant Nos. 31760648 and 31860638) and Guangxi Natural Science Foundation (Grant No. AB16380042).

## Conflict of Interest

The authors declare that the research was conducted in the absence of any commercial or financial relationships that could be construed as a potential conflict of interest.

## Publisher's Note

All claims expressed in this article are solely those of the authors and do not necessarily represent those of their affiliated organizations, or those of the publisher, the editors and the reviewers. Any product that may be evaluated in this article, or claim that may be made by its manufacturer, is not guaranteed or endorsed by the publisher.

## Abbreviations

CRISPR: Clustered regularly interspaced short palindromic repeats
sgRNAs: small guide RNA
FBS: Calf Serum
DMEM: Dulbecco's modified eagle medium.
