# Supplementary material for: Generation of Heritable Prominent Double Muscle Buttock Rabbits via Novel Site Editing of Myostatin Gene Using CRISPR/Cas9 System
Source: Front Vet Sci. 2022 May 20;9:842074. doi: 10.3389/fvets.2022.842074 (PMC9165342; doi:10.3389/fvets.2022.842074)
Supplement: Supplementary file 1 [file Data_Sheet_1.docx]

Table S1. The rabbits body weight (g) data of F0 generation

| Age/Day | M2-1 | M15-2 | T2-2 | T7-1 | T7-4 | WT1 | WT2 | T2-3（WT） | T7-2（WT） | T7-3（WT） | |
| --- | --- | --- | --- | --- | --- | --- | --- | --- | --- | --- | --- |
| 1 | 88 | 85.4 | 72.5 | 80.6 | 58.7 | 74.2 | 81.5 | 73 | 76.8 | 70.1 |  |
| 20 | 326 | 360 | 430 | 490 | 310 | 459 | 431 | 410 | 460 | 480 |  |
| 40 | 1090 | 1200 | 1280 | 1300 | 1228 | 1132 | 1090 | 1250 | 1190 | 1148 |  |
| 60 | 1900 | 1930 | 2100 | 1890 | 1810 | 2130 | 1940 | 1960 | 1670 | 1800 |  |
| 80 | 2510 | 2660 | 2415 | 2285 | 2390 | 2656 | 2595 | 2385 | 2080 | 2250 |  |
| 100 | 3119 | 2980 | 2790 | 2585 | 2820 | 3120 | 2940 | 2630 | 2275 | 2340 |  |
| 120 | 3520 | 3300 | 3200 | 2780 | 2950 | 3180 | 2980 | 2940 | 2410 | 2580 |  |
| 140 | 3448 | 3670 | 3430 | 2960 | 3130 | 2960 | 3200 | 2980 | 2630 | 2750 |  |
| 160 | 3580 | 3830 | 3690 | 3480 | 3380 | 3060 | 3340 | 3100 | 2910 | 3060 |  |
| 180 | 3660 | 3760 | 3820 | 3730 | 3570 | 3120 | 3380 | 3250 | 3120 | 3250 |  |

Table S2. The rabbits body weight (g) data of F1 generation

| Age/Day | A7-5 | A9-5 | A10-1 | A7-1 | B2-3 | A11-3 (WT) | A7-2 (WT) | A7-6 (WT) | A2-6 (WT) | B6-1 (WT) | A1-1 | A2-4 | A3-2 | B3-1 | B-2 |
| --- | --- | --- | --- | --- | --- | --- | --- | --- | --- | --- | --- | --- | --- | --- | --- |
| 1 | 58.7 | 64 | 57.5 | 52.6 | 65.2 | 52 | 62.5 | 70 | 59 | 58 | 62.9 | 57.3 | 64.2 | 60 | 57.8 |
| 20 | 480 | 240 | 450 | 440 | 374 | 390 | 390 | 430 | 410 | 380 | 450 | 440 | 460 | 376 | 345 |
| 40 | 1190 | 590 | 1080 | 1280 | 1250 | 1210 | 1210 | 1120 | 1090 | 1040 | 1290 | 1190 | 1090 | 1120 | 1250 |
| 60 | 1820 | 1350 | 1810 | 1970 | 1740 | 1890 | 1890 | 1790 | 1770 | 1740 | 1790 | 2000 | 1860 | 1810 | 1850 |
| 80 | 2100 | 2020 | 2100 | 2310 | 2600 | 2300 | 2000 | 1980 | 2040 | 2000 | 2280 | 2140 | 1930 | 2520 | 2460 |
| 100 | 2440 | 2310 | 2680 | 2600 | 3360 | 2700 | 2160 | 2260 | 2160 | 2350 | 2480 | 2680 | 2240 | 3020 | 2900 |
| 120 | 2945 | 2680 | 3220 | 3090 | 3700 | 2930 | 2580 | 2810 | 2460 | 2800 | 2780 | 3000 | 2780 | 3600 | 3400 |
| 140 | 3280 | 3570 | 3660 | 3360 | 3900 | 3200 | 2900 | 3160 | 2840 | 3220 | 3140 | 3350 | 3280 | 3600 | 3620 |
| 160 | 3860 | 4100 | 3920 | 3640 | 3920 | 3350 | 3240 | 3540 | 3040 | 3340 | 3380 | 3640 | 3340 | 3680 | 3600 |
| 180 | 4150 | 4330 | 4100 | 3880 | 4020 | 3310 | 3560 | 3830 | 3440 | 3475 | 3660 | 3640 | 3670 | 3780 | 3720 |
